# Measuring human behavior through controlled perturbations: a framework for reconstructing behavioral systems

**DOI:** 10.3389/fpsyg.2026.1833113

**Published:** 2026-08-10

**Authors:** Pietro Cipresso

**Affiliations:** Department of Psychology, University of Turin, Turin, Italy

**Keywords:** behavioral measurement, behavioral trajectories, computational psychometrics, dynamical systems, experimental design, perturbation-based experiments, quantitative psychology, system identification

## Abstract

Human behavior is typically measured by relating observed responses to latent traits inferred from statistical models. Although this approach has produced powerful tools for psychological assessment, it largely treats behavior as a static property of individuals rather than as the observable output of an evolving dynamical system. Yet most behavioral phenomena emerge from continuous interactions between internal states and environmental conditions, generating trajectories of actions, decisions, and physiological responses over time. Here we propose a framework in which human behavior is measured by reconstructing the dynamical systems that generate it through controlled perturbations. In this perspective, experimental environments act as measurement instruments that probe behavioral dynamics through structured perturbations. Behavioral observations are interpreted as the outputs of stochastic controlled dynamical systems, and behavioral measurement becomes a system identification problem: generative models are reconstructed so as to reproduce the trajectory distributions produced by human behavior under equivalent perturbation conditions. We formalize this framework using stochastic dynamical systems, introduce criteria for reconstruction and functional equivalence under unseen perturbations, and analyze the roles of perturbation design, identifiability, and experimental domain boundaries. The approach is illustrated across multiple behavioral domains, including affective dynamics, cognitive flexibility, stress reactivity, and social interaction. By integrating controlled perturbations, dynamical modeling, and trajectory-level validation, this framework reframes behavioral measurement as the reconstruction of generative behavioral dynamics. More broadly, it suggests a path toward a metrology of human behavior in which programmable experimental environments, such as immersive virtual reality platforms, serve as instruments capable of systematically probing and characterizing behavioral systems.

## Introduction: the measurement problem in behavioral science

Human behavior is widely studied across the behavioral sciences, yet the question of how behavior should be measured remains largely unresolved (Borsboom, 2005). From psychology and neuroscience to economics and human–computer interaction, researchers seek to identify the processes that generate observable behavioral patterns, an objective that has also been addressed in probabilistic and generative frameworks such as the free-energy principle (Friston, 2010). Yet despite this common goal, most measurement frameworks primarily focus on internal generative models and treat behavior as a static property of individuals rather than as the observable output of a dynamical system evolving over time, an idea that has long been emphasized in dynamical approaches to cognition and behavior (Molenaar, 2004; Van Gelder, 1995; Thelen and Smith, 1994).

Human behavior unfolds through continuous interactions (Kelso, 1995) between internal states and environmental conditions, giving rise to temporally structured patterns that have been extensively studied within dynamical systems frameworks (Kelso, 1995). Capturing these dynamics requires measurement approaches that move beyond static latent traits toward the reconstruction of the systems that generate behavioral trajectories (Hamaker and Wichers, 2017; Hamaker et al., 2018; Kuppens et al., 2010; Speyer et al., 2024).

Early psychometric theory already recognized the importance of establishing a rigorous measurement framework for behavioral phenomena. In particular, Rasch emphasized that measurement in the behavioral sciences should aim to establish invariant relations between observations and latent variables, independent of the particular sample of items or persons used in a study.

One reason for this discrepancy lies in a ™longstanding methodological constraint in behavioral research. Experimental paradigms have historically faced a trade-off between ecological validity and experimental control (Bronfenbrenner, 1977; Pooja et al., 2024). Laboratory experiments provide high levels of control and repeatability but typically rely on simplified tasks that capture only limited aspects of real-world behavior. In contrast, naturalistic observation and experience sampling methods allow researchers to study behavior in realistic contexts but provide little control over the perturbations experienced by participants (Shiffman et al., 2008; Csikszentmihalyi and Larson, 2014; Kahneman et al., 2004; Fritz et al., 2024; Xie et al., 2024). As a result, behavioral measurement has often relied on statistical inference from observational data rather than on experimental probing of behavioral systems.

Recent technological developments are beginning to alter this landscape. Advances in immersive experimental environments, multimodal sensing, and computational modeling make it increasingly feasible to observe rich behavioral trajectories while maintaining experimental control over environmental conditions (Bohil et al., 2011; Cipresso, 2015; Slater and Sanchez-Vives, 2016; Faria et al., 2023; Quirós-Ramírez et al., 2025). In particular, immersive environments, such as those enabled by virtual reality technologies, allow researchers to construct programmable experimental contexts in which environmental perturbations can be systematically manipulated while preserving a high degree of experiential realism. At the same time, modern sensing technologies allow the continuous recording of behavioral signals such as movement, gaze, physiological responses, and interaction patterns (Bohil et al., 2011; Potter et al., 2023).

These developments open the possibility of approaching behavioral measurement from a different perspective. Instead of treating behavioral observations as isolated responses from which latent traits are inferred, behavioral systems can be studied by systematically probing them with controlled perturbations and analyzing the resulting behavioral trajectories. This approach closely resembles the logic of system identification in engineering and control theory, where the goal is to reconstruct system dynamics from input–output data through carefully designed inputs (Ljung, 2010; Bellman and Åström, 1970; Söderström and Stoica, 1988).

The framework developed here aims to provide a conceptual foundation for a more dynamical and experimentally grounded approach to behavioral measurement. We first formalize behavioral systems as stochastic dynamical processes interacting with controlled perturbations. We then introduce criteria for reconstructing generative models from perturbation–response trajectories and for evaluating functional equivalence between reconstructed models and behavioral systems. Finally, we discuss how this perspective relates to existing traditions in psychometrics and computational modeling and how it may contribute to the development of a metrology of human behavior (Cipresso and Immekus, 2017).

In this work, we introduce a perturbation-based framework for reconstructing behavioral systems from trajectory data generated under controlled environmental perturbations. By integrating controlled perturbations, dynamical modeling, and trajectory-level validation, the proposed approach reframes behavioral measurement as a system identification problem. We formalize the framework, illustrate its implementation across behavioral domains, and discuss its implications for psychometrics, computational modeling, and the development of a metrology of human behavior.

## The limits of current measurement paradigms

Despite substantial progress in the behavioral sciences, the problem of how to measure human behavior remains only partially resolved. Existing approaches have produced powerful tools for quantifying individual differences and for modeling observed responses, yet they are constrained by fundamental methodological limitations that restrict their ability to capture the dynamical nature of behavior.

A central feature of most traditional measurement frameworks is the reliance on latent variables inferred from patterns of observed responses. In psychometrics, models such as factor analysis and item response theory establish probabilistic relationships between latent traits and observable outcomes, enabling the estimation of individual differences along relatively stable dimensions (Borsboom, 2005; Cronbach and Meehl, 1955). While these approaches have achieved high levels of reliability and scalability, they implicitly treat behavior as a static or slowly varying property of individuals. Temporal structure is often reduced to aggregated scores, and the underlying processes that generate behavioral trajectories remain largely unmodeled.

Parallel developments in ecological and ambulatory assessment have attempted to overcome this limitation by capturing behavior in naturalistic contexts. Experience sampling methods and related approaches provide access to behavior as it unfolds in daily life, increasing ecological validity and enabling the study of within-person variability (Kahneman et al., 2004; Shiffman et al., 2008; Fritz et al., 2024). However, these methods introduce a different limitation: the absence of experimental control over the perturbations experienced by individuals. Because environmental inputs are neither controlled nor systematically varied, it becomes difficult to disentangle the causal structure underlying observed behavioral trajectories. As a result, inference remains largely observational, and the identification of generative mechanisms is limited.

Laboratory-based experimental paradigms occupy an intermediate position, offering precise control over stimuli and high levels of repeatability. Controlled tasks allow researchers to manipulate specific variables and test well-defined hypotheses, often with fine temporal resolution. Yet this control is typically achieved at the cost of ecological validity. Simplified tasks capture only restricted aspects of real-world behavior, and the resulting measurements may fail to generalize beyond the experimental setting. Consequently, laboratory paradigms often provide high internal validity but limited insight into the broader dynamical structure of behavior in natural environments (Bohil et al., 2011; Bronfenbrenner, 1977; Cipresso, 2015; Faria et al., 2023).

These three approaches reflect a longstanding trade-off in behavioral research between ecological validity and experimental control. Naturalistic methods capture realistic behavior but lack controllability, while laboratory experiments provide control but at the expense of realism. Traditional psychometric models, in turn, offer scalable measurement but abstract away from temporal dynamics and environmental interaction. None of these paradigms simultaneously provides controlled, repeatable, and ecologically meaningful observations of behavioral trajectories (Shiffman et al., 2008; Bronfenbrenner, 1977).

This limitation has important consequences for the development of a rigorous measurement science of behavior. Without controlled perturbations, it is difficult to treat behavioral systems as objects that can be systematically probed and identified (Åström, 1995; Åström and Eykhoff, 1971). Without temporally rich observations, it is difficult to characterize the dynamical processes underlying behavioral change. Without reproducible experimental conditions, it is difficult to establish invariance and comparability across studies.

The result is that behavioral measurement remains largely indirect. Rather than identifying the mechanisms that generate behavior, current approaches typically infer latent structures from observed data or describe statistical regularities in behavioral patterns. While these strategies have proven useful, they do not fully address the challenge of reconstructing the generative systems that produce behavior.

These limitations motivate the need for a different measurement paradigm. A framework that integrates controlled perturbations, temporally extended observations, and reproducible experimental environments would make it possible to treat behavioral systems as dynamical entities that can be systematically interrogated. Such a framework would move beyond the estimation of static traits and toward the reconstruction of the processes that generate behavioral trajectories (Hamaker and Wichers, 2017; Molenaar, 2004; Speyer et al., 2024).

## A perturbation-based perspective on behavioral measurement

The limitations of current measurement paradigms suggest the need for a shift in how behavioral phenomena are conceptualized and measured. Rather than treating behavior as a static property to be inferred from observed responses, it may be more appropriate to view behavior as the observable output of a dynamical system interacting with its environment. Within this perspective, measurement is not a passive process of observation, but an active process of system interrogation (Ljung, 2010).

In many areas of science, the identification of dynamical systems relies on the application of controlled inputs, whose design critically determines the informativeness of the resulting data (Gevers and Ljung, 1986). This principle is central to system identification, where the properties of an unknown system are inferred by probing it with structured perturbations. Extending this idea to behavioral science suggests that meaningful measurement requires not only observing behavior, but actively shaping the conditions under which behavior unfolds.

From this viewpoint, environmental conditions can be interpreted as inputs to a behavioral system, while observable actions, decisions, and physiological responses constitute its outputs. Behavioral trajectories emerge from the continuous interaction between these inputs and the internal dynamics of the system. Measurement, therefore, becomes the problem of reconstructing the system that generates these trajectories, rather than summarizing the trajectories themselves. A key implication of this perspective is that perturbations are not merely experimental manipulations introduced to test specific hypotheses, but constitute the core mechanism through which behavioral systems can be identified. Carefully designed perturbations determine the informativeness of the resulting data, enabling the experimenter to explore the space of possible system responses and reveal aspects of the underlying dynamics that would remain unobservable under passive observation. The informativeness of measurement is thus directly linked to the structure and diversity of the perturbations applied.

This shift also redefines the role of experimental environments. Instead of serving solely as contexts in which behavior is observed, environments become measurement instruments that implement controlled perturbations. Advances in programmable and immersive environments, including VR-based systems, make it increasingly feasible to construct experimental settings that are both ecologically meaningful and precisely controllable. These environments enable the systematic exploration of behavioral dynamics under reproducible conditions (Bohil et al., 2011; Slater and Sanchez-Vives, 2016; Cipresso, 2015; Cipresso et al., 2018; Chirico et al., 2016; Riva, 2009; Riva et al., 2019).

Within this framework, the goal of behavioral measurement is to reconstruct a model of the system that is capable of reproducing the observed trajectories under a range of perturbations. Importantly, the validity of such a model cannot be established solely by its ability to fit observed data, but must be evaluated based on its capacity to generate behavior that is indistinguishable from that of the original system when exposed to new perturbations. This notion of functional equivalence shifts the focus from descriptive adequacy to generative fidelity (Box, 1976).

Adopting a perturbation-based perspective thus transforms behavioral measurement into a problem of dynamical system reconstruction under controlled experimental conditions. This perspective provides a unifying framework that integrates experimental design, data collection, and modeling into a single process, and sets the stage for a more principled and reproducible science of human behavior.

## Formal framework for perturbation-based behavioral reconstruction

The central premise of the present framework is that human behavior should be understood not merely as a set of observable responses but as the manifestation of an underlying dynamical system interacting with structured environmental perturbations. In this perspective, psychological measurement is reframed from the estimation of latent scores to the reconstruction of generative behavioral dynamics. The objective is therefore not simply to predict responses but to identify a model capable of reproducing the trajectories produced by a behavioral system when subjected to controlled interventions (Schwarz, 1978; Borel, 2012).

**Figure 1:**
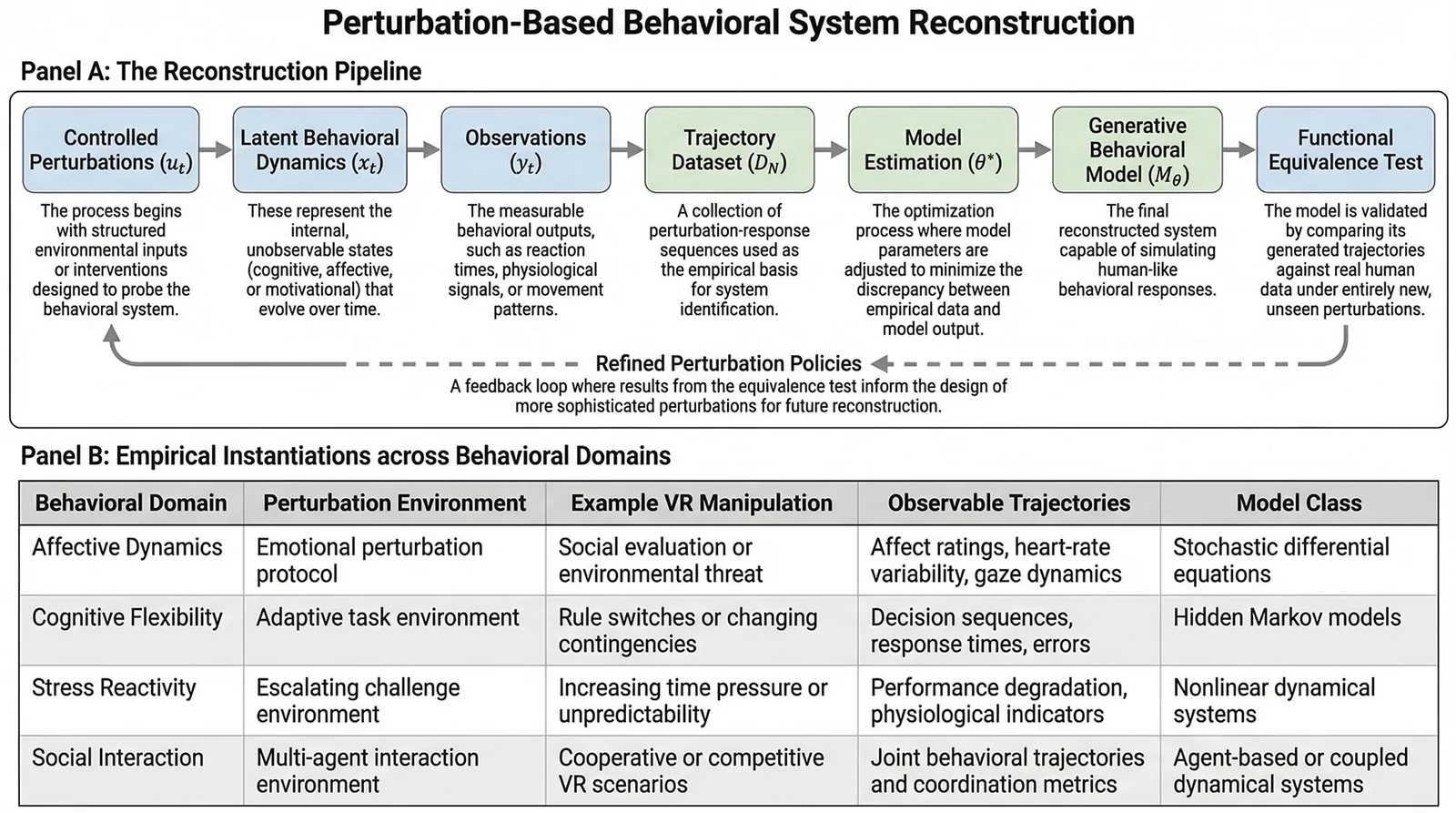
Perturbation-based behavioral system reconstruction framework. Behavioral measurement is formulated as a system identification problem in which controlled perturbations *u*_*t*_ probe latent behavioral states *x*_*t*_, producing observable trajectories *y*_*t*_. These perturbation–response trajectories form a dataset *D*_*N*_used to estimate a generative behavioral model $M_{\theta }$. Reconstruction is validated through functional equivalence tests evaluating whether the model generates trajectory distributions indistinguishable from those produced by the human system under previously unseen perturbations. The validation process informs the design of refined perturbation policies, forming an iterative experimental loop. The lower panel illustrates representative behavioral domains and corresponding model classes that can instantiate the reconstruction framework.

The overall structure of the perturbation-based behavioral reconstruction framework is summarized in Figure 1. The framework conceptualizes behavioral measurement as a system identification process in which controlled perturbations probe latent behavioral dynamics, generating trajectory data used to reconstruct and validate generative models of behavior.

To formalize this idea, we represent a behavioral system as a stochastic controlled dynamical system (Oksendal, 2013; Pavliotis, 2014). Let *x*_*t*_ denote the latent behavioral state of an individual at time, *u*_*t*_ a perturbation applied by the experimental environment, and *y*_*t*_ the observable behavioral output. The evolution of the system is described by the pair of equations


$$\begin{array}{l} x_{t+1}\sim Q\left(\cdot |x_{t},u_{t}\right),\;y_{t}\sim R\left(\cdot |x_{t}\right), \end{array}$$

where *Q* is a transition kernel describing how the internal state evolves under perturbations and *R* is an observation kernel mapping latent states to measurable outputs. The state *x*_*t*_ may represent cognitive, affective, or motivational variables that are not directly observable, whereas the output *y*_*t*_ may correspond to reaction times, movement trajectories, physiological signals, or affective reports.

Given a sequence of perturbations *u*_0:*T*−1_ = (*u*_0_, …, *u*_*T*−1_), the behavioral system induces a probability distribution over observable trajectories *y*_0:*T*_ = (*y*_0_, …, *y*_*T*_). We denote this trajectory law by


$$\begin{array}{l} P_{B}\left(y_{0:T}|u_{0:T-1}\right), \end{array}$$

where B refers to the underlying behavioral system. This distribution constitutes the fundamental object of measurement within the present framework. Unlike classical psychometric approaches that summarize behavior through static scores or averaged responses, the trajectory law captures the full temporal evolution of behavior under controlled perturbation.

As illustrated in Figure 1, the perturbation sequence *u*_*t*_ acts as an external input probing the latent behavioral state *x*_*t*_, while the observable outputs *y*_*t*_ correspond to measurable behavioral trajectories generated by the underlying dynamical system.

A central but often implicit assumption in behavioral modeling concerns the unit of reconstruction. In the present framework, two distinct formulations must be distinguished. In the idiographic formulation, the goal is to reconstruct the dynamics of a single behavioral system *B*^(*i*)^, corresponding to an individual. The dataset then consists of multiple perturbation-response trajectories collected from the same system under varying conditions:


$$\begin{array}{l} D_{N}={\left\{\left(u_{0:T_{k}-1}^{\left(k\right)},y_{0:T_{k}}^{\left(k\right)}\right)\right\}}_{k=1}^{N} \end{array}$$

In this setting, reconstruction corresponds to identifying a generative model that approximates the individual-specific trajectory law $P_{B^{\left(i\right)}}\left(y_{0:T}|u_{0:T-1}\right)$. This formulation implicitly requires assumptions about the stability or ergodicity of the underlying system across trials.

In contrast, a nomothetic formulation considers a population of behavioral systems, where each trajectory may originate from a different individual. In this case, the objective is to reconstruct a population-level trajectory distribution:


$$\begin{array}{l} P\left(y_{0:T}|u_{0:T-1}\right)=\int P_{B}\left(y_{0:T}|u_{0:T-1}\right)dP\left(B\right) \end{array}$$

While this formulation aligns more closely with classical psychometric approaches, it fundamentally alters the reconstruction problem and may obscure individual-specific dynamics. As shown by Molenaar (2004), such aggregation is not generally valid under non-ergodic conditions.

The present framework is primarily grounded in the idiographic formulation, where controlled perturbations are used to probe and reconstruct individual behavioral systems. Extensions to population-level modeling require additional assumptions and are left for future work.

Empirical behavioral data consist of a set of perturbation-response trajectories obtained across experimental trials or participants, depending on the unit of reconstruction adopted. Let


$$\begin{array}{l} D_{N}={\left\{\left(u_{0:T_{i}-1}^{\left(i\right)},y_{0:T_{i}}^{\left(i\right)}\right)\right\}}_{i=1}^{N} \end{array}$$

denote a dataset containing *N* such trajectories. The goal of behavioral reconstruction is to identify a generative model capable of reproducing the trajectory law induced by the true system.

**To illustrate the operationalization of the proposed framework**, consider a cognitive flexibility task implemented in a VR environment in which participants are exposed to rule-switching perturbations. At each time step, the perturbation *u*_*t*_ corresponds to changes in task contingencies, while the observable output *y*_*t*_ consists of action choices and reaction times. A candidate model *M*_θ_, such as a switching state-space model, is fitted to a set of perturbation-response trajectories by minimizing the discrepancy between empirical and model-generated trajectory distributions. Functional equivalence is then evaluated by exposing both the participant and the reconstructed model to novel perturbation sequences not used during estimation and comparing the resulting trajectory distributions. This example illustrates how controlled perturbations, trajectory-level data, and generative modeling are integrated within a concrete experimental setting.

To this end, we consider a family of candidate models


$$\begin{array}{l} 𝔐=\left\{M_{\theta }:\theta \in \Theta \right\}, \end{array}$$

where each model $M_{\theta }$ is itself a stochastic controlled dynamical system parameterized by θ. Reconstruction consists in estimating the parameter value θ that best reproduces the empirical perturbation–response data. Formally, this can be expressed as the optimization problem


$$\begin{array}{l} \theta^{⋆}=arg\underset{\theta \in \Theta }{\min}L_{N}\left(\theta \right), \end{array}$$

where the loss function


$$\begin{array}{l} L_{N}\left(\theta \right)=\frac{1}{N}\overset{N}{\underset{i=1}{\sum }}D\left(P_{emp}^{\left(i\right)},P_{M_{\theta }}\left(\cdot |u^{\left(i\right)}\right)\right)+\lambda \Omega \left(\theta \right) \end{array}$$

quantifies the discrepancy between empirical trajectory distributions and those generated by the model. The function *D* measures the distance between trajectory distributions, while Ω(θ) imposes structural constraints or regularization on the model parameters (Cover and Thomas, 2012).

Crucially, reconstruction claims must be evaluated within explicitly defined experimental boundaries. Behavioral systems are embedded in complex environments, and it is neither meaningful nor feasible to reconstruct their dynamics universally. Instead, reconstruction is defined relative to a bounded experimental domain


$$D=(U_{D},Y_{D},T_{D},\Pi_{D}),$$

where $U_{D}$ specifies the admissible perturbations, $Y_{D}$ the admissible output space, $T_{D}$ the temporal horizon, and $\Pi_{D}$ the class of perturbation policies allowed within the experiment. All statements about reconstruction or equivalence are therefore restricted to the domain D.

Within this bounded domain, the central question becomes whether a reconstructed model can reproduce the behavior of the true system under perturbations that were not used during estimation. Let $\Pi_{D}^{train}$ denote the perturbation sequences used during reconstruction and $\Pi_{D}^{test}$ a disjoint set of perturbations reserved for validation. A reconstructed model $M_{\hat{\theta }}$ is said to be functionally equivalent to the behavioral system if the trajectory distributions generated by the two systems remain sufficiently close under all unseen perturbations in the test set. Formally, this condition can be written as


$$\begin{array}{l} \underset{u\in \Pi_{D}^{test}}{\sup}D\left(P_{B}\left(\cdot |u\right),P_{M_{\hat{\theta }}}\left(\cdot |u\right)\right)\leq \delta , \end{array}$$

where δ represents an admissible tolerance level. Functional equivalence thus requires that the reconstructed model reproduce the behavioral dynamics of the true system across novel perturbations rather than merely fitting the trajectories observed during training.

The discrepancy measure *D* may be defined in several ways depending on the nature of the behavioral data. When trajectories are deterministic or weakly stochastic, a simple Euclidean discrepancy between trajectories may suffice. In more complex settings involving stochastic variability and multimodal dynamics, it is preferable to compare probability distributions over trajectories. In such cases, a Wasserstein metric (Villani, 2009; Santambrogio, 2015) or kernel-based discrepancy such as maximum mean discrepancy provides a principled way to measure distances between path distributions (Gretton et al., 2012; Muandet et al., 2017; Schölkopf and Smola, 2002).

However, this definition relies on the comparison of trajectory laws, which are not directly observable in practice, as empirical data consist only of finite samples of trajectories collected under specific perturbations.

Accordingly, functional equivalence must be formulated as a statistical indistinguishability problem. For a given perturbation *u*, let ${\left\{y^{\left(i\right)}\right\}}_{i=1}^{n}$ denote observed trajectories and ${\left\{{\overset{\sim }{y}}^{\left(j\right)}\right\}}_{j=1}^{m}$ trajectories generated by the reconstructed model. A discrepancy measure *D* can then be used to compare the two samples:


$$\begin{array}{l} {\overset{ˆ}{D}}_{n,m}\left(u\right)=D\left({\left\{y^{\left(i\right)}\right\}}_{i=1}^{n},{\left\{{\overset{\sim }{y}}^{\left(j\right)}\right\}}_{j=1}^{m}\right) \end{array}$$

Functional equivalence is then assessed by evaluating whether the empirical discrepancy remains below a tolerance threshold across a class of perturbations:


$$\begin{array}{l} \underset{u\in \Pi^{test}}{\sup}{\overset{ˆ}{D}}_{n,m}\left(u\right)\leq \delta +\epsilon_{n,m} \end{array}$$

Where ϵ_*n, m*_ accounts for statistical uncertainty due to finite sampling. In this formulation, functional equivalence corresponds to the inability to distinguish between observed and model-generated trajectories given finite data, rather than to exact equality of the underlying trajectory laws.

This formulation naturally leads to the notion of reconstructability. A behavioral system is said to be reconstructable within a bounded experimental domain if there exists a model within the candidate class capable of achieving functional equivalence under unseen perturbations. Let 𝔐 denote the chosen model class and δ the admissible tolerance level. The behavioral system B is reconstructable on domain D if there exists θ^⋆^∈Θ such that


$$\begin{array}{l} \underset{u\in \Pi_{D}^{test}}{\sup}D\left(P_{B}\left(\cdot |u\right),P_{M_{\theta^{⋆}}}\left(\cdot |u\right)\right)\leq \delta . \end{array}$$

The minimal achievable discrepancy


$$\varepsilon^{⋆}(M,D)=\underset{\theta \in \Theta }{\inf}\underset{u\in \Pi_{D}^{test}}{\sup}D\text{​}(P_{B}(\cdot |u),P_{M_{\theta }}(\cdot |u)).$$

defines the intrinsic reconstruction error associated with the chosen model class and experimental domain. If ε^⋆^ exceeds the tolerance level, the behavioral system cannot be reconstructed within the specified domain using the given model class. Importantly, such failures are informative: they reveal structural limits on reconstructability and guide the design of richer perturbation environments or more expressive model families.

In this framework, perturbations play a central methodological role. Rather than serving merely as experimental stimuli, perturbations act as identification inputs that probe the internal dynamics of the behavioral system. The informativeness of a perturbation family can therefore be characterized by its ability to discriminate between alternative models. Let $\Pi_{D}$ denote the admissible perturbation set. Its discriminatory power can be defined as


$$\begin{array}{l} \Delta \left(\Pi_{D}\right)=\underset{\theta_{1}\neq \theta_{2}}{\inf}\underset{u\in \Pi_{D}}{\sup}D\left(P_{M_{\theta_{1}}}\left(\cdot |u\right),P_{M_{\theta_{2}}}\left(\cdot |u\right)\right). \end{array}$$

A perturbation family is informative if $\Delta \left(\Pi_{D}\right)>0$, meaning that distinct models produce distinguishable trajectory distributions under at least one admissible perturbation. This definition highlights the importance of experimental design: reconstruction is possible only if the perturbation environment generates sufficiently rich behavioral responses to reveal the underlying dynamics.

Taken together, these elements define a metrological framework for behavioral science grounded in system identification and controlled perturbation. Within bounded experimental domains, behavioral measurement becomes the problem of reconstructing generative dynamical models whose responses to unseen perturbations remain indistinguishable from those produced by the human system itself. This perspective extends the scope of psychometrics from the estimation of latent variables to the reconstruction of behavioral dynamics and provides a formal basis for next-generation computational measurement approaches.

## Perturbation environments and behavioral system emulation

The formal framework introduced above places controlled perturbations at the center of behavioral reconstruction. The ability to identify generative behavioral dynamics depends critically on the richness and controllability of the perturbation space through which the system is probed. In practical terms, this requirement translates into the need for experimental environments capable of delivering structured and reproducible interventions while simultaneously capturing high-resolution behavioral trajectories.

Traditional psychological experiments typically manipulate a limited set of discrete stimuli while measuring responses through aggregated outcomes such as accuracy rates or response times. Although such paradigms have proven valuable for testing specific hypotheses, they provide only sparse information about the underlying dynamical structure of behavior. The reconstruction framework described here instead requires environments in which perturbations can be continuously parameterized, temporally structured, and systematically varied in order to explore the response surface of the behavioral system.

Let *u*_*t*_ denote the perturbation applied to the system at time *t*. In many classical experimental paradigms, *u*_*t*_ belongs to a small finite set corresponding to categorical stimulus conditions. In contrast, perturbation-based reconstruction assumes that the perturbation space U may be multidimensional and continuously parameterized. For example, perturbations may correspond to variations in environmental complexity, cognitive load, social interaction dynamics, uncertainty levels, or temporal constraints. The perturbation sequence *u*_0:*T*−1_ therefore defines a trajectory through the perturbation space that elicits a corresponding behavioral trajectory.

A perturbation environment can thus be viewed as a mapping


$$\begin{array}{l} \Phi :\left(s_{t},u_{t}\right)\to s_{t+1}, \end{array}$$

where *s*_*t*_ denotes the state of the external environment and *u*_*t*_ represents an intervention applied to that environment. The behavioral system interacts with the environment through a perception–action loop: the environment generates stimuli affecting the internal behavioral state *x*_*t*_, while the individual's actions modify the environment itself. In this sense, the behavioral system and the environment together form a coupled dynamical system whose observable trajectories depend jointly on internal dynamics and external perturbations.

From the standpoint of reconstruction, the environment serves a dual role. First, it generates the perturbation signals required to probe the behavioral system. Second, it provides the measurement infrastructure through which behavioral outputs are observed. Ideally, the environment should therefore satisfy three properties: controllability of perturbations, observability of behavioral responses, and reproducibility of experimental conditions.

Controllability refers to the ability to manipulate perturbations across a sufficiently rich set of conditions. Observability refers to the capacity to measure behavioral outputs with sufficient temporal and spatial resolution. Reproducibility ensures that perturbation sequences can be repeated across participants or experimental sessions, allowing the trajectory distributions induced by different perturbations to be estimated reliably.

Virtual environments are particularly well-suited to meet these requirements. A virtual environment can be regarded as a programmable perturbation generator in which environmental parameters can be manipulated in real time while maintaining precise control over experimental conditions. Because the environment is generated computationally, perturbations can be defined along multiple continuous dimensions and dynamically adapted during the interaction.

Formally, let U denote the perturbation space and let $\Pi_{D}$ be the set of admissible perturbation policies defined within the bounded experimental domain introduced earlier. A virtual environment implements a perturbation policy


$$\begin{array}{l} u_{t}=\pi \left(s_{t},t\right), \end{array}$$

where $\pi \in \Pi_{D}$ determines how perturbations evolve as a function of environmental state and time. This formulation allows perturbations to depend not only on predetermined experimental schedules but also on the evolving behavior of the participant. Adaptive perturbation policies are particularly valuable for system identification because they can actively explore regions of the state space that are most informative for distinguishing competing models.

The second critical property of perturbation environments concerns observability. Modern experimental platforms allow the simultaneous recording of multiple behavioral signals, including motor trajectories, gaze patterns, physiological responses, and interaction dynamics. Let


$$\begin{array}{l} y_{t}=\left(y_{t}^{\left(1\right)},y_{t}^{\left(2\right)},\ldots ,y_{t}^{\left(k\right)}\right) \end{array}$$

$$\begin{array}{l} y_{t}\sim R\left(\cdot |x_{t}\right), \end{array}$$

therefore represents a multimodal measurement process in which latent behavioral states generate observable signals across multiple sensory channels.

The integration of multiple observation modalities improves the identifiability of behavioral dynamics. If distinct latent states produce indistinguishable outputs in a single modality, additional modalities may reveal differences that enable model discrimination. From an information-theoretic perspective, multimodal sensing increases the effective observability of the latent state space.

A third essential property of perturbation environments is reproducibility. Because virtual environments are defined computationally, the same perturbation sequence can be reproduced exactly across different experimental sessions. This reproducibility ensures that the trajectory distribution


$$\begin{array}{l} P_{B}\left(y_{0:T}|u_{0:T-1}\right) \end{array}$$

can be estimated consistently across participants exposed to identical perturbation policies. Reproducibility also allows the separation of training and testing perturbation sets required for evaluating functional equivalence. Perturbation sequences used to reconstruct the model can be strictly separated from those used to validate the reconstructed system under unseen conditions.

Within this framework, virtual environments act as behavioral system emulators. They generate controlled perturbations, record behavioral trajectories, and maintain a fully observable record of environmental dynamics. The environment therefore provides the experimental substrate on which reconstruction algorithms operate. Behavioral trajectories collected within such environments can be interpreted as realizations of the perturbation–response mapping


$$\begin{array}{l} u_{0:T-1}\mapsto y_{0:T}. \end{array}$$

Reconstruction then amounts to identifying a generative model capable of reproducing this mapping across perturbation policies within the bounded experimental domain.

Importantly, the role of the environment is not limited to delivering stimuli. By systematically exploring the perturbation space, the environment becomes an instrument for probing the internal dynamics of behavior. Carefully designed perturbation policies can reveal non-linear responses, state-dependent transitions, and resilience limits that would remain hidden under static experimental conditions. In this sense, perturbation environments perform a function analogous to experimental apparatus in the physical sciences: they provide the means through which the underlying structure of the system becomes observable.

This perspective suggests a shift in the role of experimental design within behavioral science. Rather than focusing solely on hypothesis testing, experimental environments should be engineered to maximize the informational value of perturbations for system identification. The informativeness measure introduced in the previous section,


$$\begin{array}{l} \Delta \left(\Pi_{D}\right), \end{array}$$

provides a formal criterion for evaluating the discriminatory power of different perturbation families. Perturbation environments that generate highly discriminative trajectories improve the feasibility and accuracy of behavioral reconstruction.

Consequently, the design of perturbation environments becomes a central methodological component of computational behavioral measurement. By integrating programmable perturbations, multimodal sensing, and reproducible experimental conditions, virtual environments provide the infrastructure necessary to implement the reconstruction framework described in the preceding section. They enable behavioral measurement to move beyond static observation toward an active interrogation of the dynamical mechanisms generating human behavior.

## Functional equivalence and validation

The reconstruction framework described in the previous sections raises a fundamental methodological question: how can one determine whether a reconstructed model faithfully captures the behavioral dynamics of the system under investigation? Traditional model evaluation in behavioral science typically relies on predictive accuracy or goodness-of-fit metrics computed on the data used for model estimation. However, such criteria are insufficient in the context of behavioral system reconstruction. A model may reproduce the trajectories observed during estimation without capturing the generative structure that produced them. True reconstruction requires demonstrating that the model behaves like the system itself when subjected to perturbations beyond those used during training.

For this reason, validation within the present framework is defined in terms of functional equivalence under novel perturbations. Rather than evaluating whether a model fits observed data, we evaluate whether the model generates trajectories that are statistically indistinguishable from those produced by the behavioral system when exposed to previously unseen perturbations.

Let $\Pi_{D}^{train}$ denote the family of perturbation policies used to collect the training data for reconstruction, and let $\Pi_{D}^{test}$ denote a disjoint set of perturbations reserved for validation. The key requirement is that the perturbations in the test set are not used during model estimation, thereby providing a genuine out-of-distribution evaluation of the reconstructed model.

Given a perturbation sequence $u_{0:T-1}\in \Pi_{D}^{test}$, the true behavioral system generates a trajectory distribution


$$\begin{array}{l} P_{B}\left(y_{0:T}|u_{0:T-1}\right), \end{array}$$

while the reconstructed model $M_{\hat{\theta }}$ generates its own trajectory distribution


$$\begin{array}{l} P_{M_{\hat{\theta }}}\left(y_{0:T}|u_{0:T-1}\right). \end{array}$$

The degree to which the reconstructed model captures the behavioral dynamics of the system can therefore be evaluated by comparing these two distributions. Let *D*(·, ·) denote a discrepancy measure defined over trajectory distributions (Kullback and Leibler, 1951). Functional equivalence between the reconstructed model and the behavioral system can then be expressed as the condition


$$\begin{array}{l} \underset{u\in \Pi_{D}^{test}}{\sup}D\left(P_{B}\left(\cdot |u\right),P_{M_{\hat{\theta }}}\left(\cdot |u\right)\right)\leq \delta , \end{array}$$

where δ represents a tolerance threshold reflecting measurement noise, model approximation error, and stochastic variability in behavioral trajectories.

This definition captures the essential requirement that the reconstructed model must reproduce the behavioral system's responses across a family of perturbations rather than merely under the specific conditions used for estimation. In practical terms, the model is considered successful if an external observer exposed only to the trajectory distributions generated by the model and by the real system would be unable to reliably distinguish between them.

The choice of discrepancy measure *D* depends on the nature of the behavioral trajectories and the stochastic structure of the system. In deterministic or low-noise contexts, simple pointwise discrepancies between trajectories may suffice. However, behavioral dynamics are typically stochastic and heterogeneous across individuals and trials. In such settings, comparison at the level of probability distributions over trajectories is more appropriate (Goodfellow et al., 2020). Distributional discrepancies such as the Wasserstein distance or kernel-based divergences provide principled ways to compare stochastic trajectory ensembles while accounting for variability in timing, amplitude, and path structure.

An important implication of this validation framework is that reconstruction becomes intrinsically tied to the notion of out-of-distribution generalization. A model that perfectly reproduces training trajectories may still fail when confronted with novel perturbations. Such failures reveal that the reconstructed model captures only superficial statistical regularities rather than the underlying dynamical structure of behavior. Conversely, a model that continues to generate indistinguishable trajectories under unseen perturbations provides stronger evidence that the generative mechanisms governing the behavioral system have been successfully captured.

Functional equivalence therefore shifts the focus of model validation from retrospective fit to prospective behavioral indistinguishability. This perspective is closely related to ideas from system identification and generative modeling, where models are evaluated according to their ability to reproduce the observable consequences of a system's internal dynamics. In the present context, the criterion of indistinguishability is evaluated over the space of perturbation–response trajectories rather than static outputs.

This formulation also highlights the importance of the perturbation environment in the validation process. The strength of the validation test depends on the richness of the perturbation policies included in $\Pi_{D}^{test}$. If the test perturbations explore only a narrow region of the perturbation space, models that are structurally incorrect may still appear equivalent to the behavioral system. Conversely, a diverse set of perturbations probing different regions of the behavioral state space increases the likelihood that structural discrepancies between the model and the system will become observable.

From this perspective, validation experiments can be interpreted as stress tests of the reconstructed behavioral model. Perturbations that push the system toward its dynamical boundaries—such as high cognitive load, abrupt environmental changes, or social disruptions—are particularly informative because they expose non-linearities and state transitions that may not be apparent under mild conditions. These perturbations reveal whether the reconstructed model captures not only the nominal behavior of the system but also its response to extreme or rare conditions.

The validation framework also provides a natural way to characterize reconstruction failures. Suppose that, for a given model class, the minimal achievable discrepancy between the behavioral system and any model in the class is


$$\varepsilon^{⋆}(M,D)=\underset{\theta \in \Theta }{\inf}\underset{u\in \Pi_{D}^{test}}{\sup}D\text{​}(P_{B}(\cdot |u),P_{M_{\theta }}(\cdot |u)).$$

If ε^⋆^ exceeds the tolerance level, the behavioral system cannot be reconstructed within the bounded experimental domain using the specified model class. Rather than being interpreted as a methodological failure, such outcomes reveal fundamental limits on reconstructability. They indicate either that the model class lacks sufficient expressive power to capture the dynamics of the system or that the perturbation environment fails to generate sufficiently informative trajectories.

In this way, validation becomes not merely a test of model performance but a diagnostic tool for understanding the structure of behavioral systems. Reconstruction success suggests that the system's dynamics are adequately captured within the chosen model class and perturbation domain. Reconstruction failure, on the other hand, points toward deeper complexity in the behavioral process or toward insufficient perturbation richness in the experimental environment.

Ultimately, the concept of functional equivalence establishes a rigorous criterion for behavioral system reconstruction. By requiring indistinguishability under novel perturbations, it ensures that reconstructed models capture the generative mechanisms underlying behavioral dynamics rather than simply fitting observed data. This principle provides the methodological foundation for a perturbation-based approach to behavioral measurement in which models are evaluated according to their ability to reproduce the observable consequences of human behavior across a controlled space of environmental interventions.

## Perturbation informativeness and experimental design

The reconstruction framework presented in the preceding sections emphasizes that behavioral dynamics can only be identified through sufficiently informative perturbations. While functional equivalence provides a criterion for evaluating reconstructed models, successful reconstruction depends critically on the design of perturbation policies capable of revealing the internal structure of the behavioral system. Experimental design therefore becomes a central methodological component of behavioral reconstruction.

In classical experimental psychology, experimental manipulations are typically selected to test specific hypotheses about cognitive or affective processes. Conditions are designed to isolate particular effects, often through factorial manipulations or controlled contrasts between stimulus categories. Although this strategy has been highly effective for hypothesis testing, it is not optimized for system identification. From the perspective of reconstruction, the objective is not simply to detect differences between conditions but to generate behavioral trajectories that expose the dynamical structure of the system (Strogatz, 2024).

Within the present framework, perturbations function as identification inputs analogous to those used in control theory and system identification. Let $\Pi_{D}$ denote the family of admissible perturbation policies defined within the bounded experimental domain. Each policy $\pi \in \Pi_{D}$ generates a sequence of perturbations *u*_0:*T*−1_ that interacts with the behavioral system and produces a corresponding trajectory distribution


$$\begin{array}{l} P_{B}\left(y_{0:T}|u_{0:T-1}\right). \end{array}$$

Different perturbation policies may elicit trajectories that are more or less informative about the underlying system dynamics (Strogatz, 2024). If two distinct candidate models generate indistinguishable trajectories under a given perturbation policy, that policy provides no information for discriminating between them. Conversely, perturbations that produce strongly divergent trajectories across models reveal structural differences in their underlying dynamics.

To formalize this intuition, consider two candidate models $M_{\theta_{1}}$ and $M_{\theta_{2}}$ belonging to the model class 𝔐. The ability of a perturbation sequence to discriminate between these models can be measured by the discrepancy between their induced trajectory distributions:


$$\begin{array}{l} D\left(P_{M_{\theta_{1}}}\left(\cdot |u\right),P_{M_{\theta_{2}}}\left(\cdot |u\right)\right). \end{array}$$

A perturbation family $\Pi_{D}$ is informative if, for any pair of distinct parameter values, there exists at least one perturbation policy capable of producing distinguishable behavioral trajectories. This property can be captured by defining the discriminatory power of the perturbation family as


$$\begin{array}{l} \Delta \left(\Pi_{D}\right)=\underset{\theta_{1}\neq \theta_{2}}{\inf}\underset{u\in \Pi_{D}}{\sup}D\left(P_{M_{\theta_{1}}}\left(\cdot |u\right),P_{M_{\theta_{2}}}\left(\cdot |u\right)\right). \end{array}$$

If $\Delta \left(\Pi_{D}\right)>0$, distinct models generate distinguishable trajectory distributions under at least one admissible perturbation. In such cases, the perturbation family contains sufficient information to enable reconstruction in principle. Conversely, if $\Delta \left(\Pi_{D}\right)=0$, there exist distinct models that remain indistinguishable under all admissible perturbations, making reconstruction impossible within the given experimental domain.

This formulation reveals that the feasibility of behavioral reconstruction depends not only on the choice of model class but also on the design of the perturbation environment. Even highly expressive models cannot be reliably identified if the perturbation policies fail to probe the relevant dimensions of the behavioral state space.

From this perspective, the design of perturbation environments becomes an optimization problem. Let A denote the set of feasible perturbation policies that can be implemented within the experimental infrastructure. The objective is to identify the perturbation family that maximizes discriminatory power across the model class. Formally, the optimal perturbation family can be defined as


$$\begin{array}{l} \Pi_{D}^{⋆}=arg\underset{\Pi \in A}{\max}\Delta \left(\Pi \right). \end{array}$$

Perturbation policies selected according to this criterion generate behavioral trajectories that are maximally informative for distinguishing between competing models of behavioral dynamics.

In practice, optimal perturbation design may involve balancing several competing objectives. Highly informative perturbations may also induce extreme behavioral responses that compromise ecological validity or participant comfort. Furthermore, certain perturbations may be infeasible due to experimental constraints or ethical considerations. As a result, optimal perturbation design typically involves maximizing informational value subject to feasibility and safety constraints.

An important advantage of programmable perturbation environments is their ability to implement adaptive perturbation policies. Rather than predefining a fixed sequence of perturbations, the environment can dynamically adjust perturbations in response to the evolving behavior of the participant. Let *s*_*t*_ denote the environmental state and *y*_*t*_ the observed behavioral output at time *t*. An adaptive perturbation policy can then be expressed as


$$\begin{array}{l} u_{t}=\pi \left(s_{t},y_{0:t}\right), \end{array}$$

allowing perturbations to depend on the history of observed behavior. Such policies can actively explore regions of the behavioral state space that are most informative for system identification. For example, if the current behavioral trajectory suggests that the system is near a stability boundary, the perturbation policy may introduce additional stressors to reveal non-linear transitions or resilience limits.

Adaptive perturbation design transforms the experimental environment into an active interrogation mechanism for behavioral systems. Instead of passively presenting stimuli, the environment interacts with the participant in a closed loop, continuously adjusting perturbations to maximize the informational value of the resulting trajectories.

This approach aligns behavioral experimentation with principles from optimal experimental design and active learning. By systematically selecting perturbations that maximize information gain about model parameters or system structure, the experimental process itself becomes part of the reconstruction algorithm. Data collection and model identification are therefore integrated within a unified framework.

The implications of this perspective extend beyond the design of individual experiments. Perturbation environments can be progressively refined as reconstruction progresses. Early experiments may employ broad exploratory perturbations to identify coarse features of the behavioral system. Subsequent experiments can then focus on perturbations that discriminate between competing models or refine estimates of critical dynamical parameters. In this way, the experimental design evolves iteratively alongside the reconstruction process.

Ultimately, perturbation informativeness provides a formal bridge between experimental methodology and computational modeling in behavioral science. By treating perturbations as identification inputs and by designing experimental environments that maximize their informational value, behavioral research can move beyond static observation toward an active reconstruction of the dynamical mechanisms that generate human behavior.

## Identifiability and limits of behavioral reconstruction

The reconstruction framework described in the preceding sections establishes a formal methodology for identifying behavioral systems from perturbation–response trajectories. However, the feasibility of reconstruction depends on fundamental theoretical conditions. Even in the presence of rich experimental data, it is possible that distinct models generate indistinguishable observable trajectories. In such cases the behavioral system cannot be uniquely reconstructed. Understanding the conditions under which reconstruction is possible therefore requires a careful analysis of identifiability and reconstructability limits.

Identifiability concerns whether the parameters or structures of a model can be uniquely inferred from observable data. Within the present framework, the observable data consist of trajectory distributions generated under admissible perturbation policies. Let $𝔐=\left\{M_{\theta }:\theta \in \Theta \right\}$ denote the candidate model class. Each model induces a family of trajectory distributions


$$\begin{array}{l} P_{M_{\theta }}\left(y_{0:T}|u_{0:T-1}\right) \end{array}$$

for perturbation sequences *u*_0:*T*−1_ drawn from the admissible perturbation family $\Pi_{D}$. Reconstruction is possible only if distinct parameter values generate distinguishable trajectory distributions across the perturbation space.

This condition can be formalized through the concept of structural identifiability. The model class 𝔐 is structurally identifiable on the bounded experimental domain D if


$$\begin{array}{l} P_{M_{\theta_{1}}}\left(\cdot |u\right)=P_{M_{\theta_{2}}}\left(\cdot |u\right)\;\forall u\in \Pi_{D}\;\Rightarrow \;\theta_{1}=\theta_{2}. \end{array}$$

This condition states that two parameter values producing identical trajectory distributions under all admissible perturbations must in fact be identical. If structural identifiability fails, multiple models remain observationally indistinguishable regardless of the amount of available data.

Structural identifiability is a property of the model class and perturbation environment rather than the dataset itself. Even with infinite data, reconstruction is impossible if the perturbation policies fail to reveal differences between candidate models. The informativeness criterion introduced earlier provides a complementary perspective on this issue. A perturbation family capable of separating all models within the candidate class ensures that the identifiability condition can, in principle, be satisfied.

In practice, behavioral data are finite and subject to measurement noise, individual variability, and stochastic fluctuations in behavioral trajectories. These factors give rise to the concept of practical identifiability. Even when a model class is structurally identifiable, parameter estimation may remain unstable if the available data do not provide sufficient information to constrain the parameter space.

Let ${\hat{\theta }}_{N}$ denote an estimator obtained from *N* perturbation–response trajectories. Practical identifiability requires that the uncertainty associated with the estimator decreases as the amount of data increases. Formally, the estimator should satisfy a consistency condition such as


$$\begin{array}{l} {\hat{\theta }}_{N}\to \theta^{⋆}\;as\;N\to \infty , \end{array}$$

where θ^⋆^ denotes the parameter value corresponding to the true behavioral system within the model class. When this condition holds, increasing the number or diversity of perturbation trajectories improves the accuracy of reconstruction.

However, even under ideal estimation conditions, reconstruction remains constrained by the expressive power of the model class. The true behavioral system may lie outside the candidate model family, in which case perfect reconstruction is impossible. The discrepancy between the true system and the best approximating model defines the intrinsic reconstruction error


$$\varepsilon^{⋆}(M,D)=\underset{\theta \in \Theta }{\inf}\underset{u\in \Pi_{D}}{\sup}D\text{​}(P_{B}(\cdot |u),P_{M_{\theta }}(\cdot |u)).$$

This quantity represents the smallest achievable discrepancy between the behavioral system and any model within the candidate class over the admissible perturbation domain. If ε^⋆^ exceeds the tolerance level required for functional equivalence, the behavioral system cannot be reconstructed within the specified model class.

The concept of intrinsic reconstruction error highlights a fundamental trade-off in behavioral modeling. Model classes with limited complexity may fail to capture essential dynamical features of the system, leading to large reconstruction errors. Conversely, highly expressive model classes may introduce identifiability challenges, as many parameter configurations may generate similar observable trajectories. Effective reconstruction therefore requires balancing expressive power with identifiability constraints.

An additional limitation arises from the bounded nature of experimental domains. Behavioral systems operate across a vast range of environmental conditions, many of which cannot be reproduced experimentally. Reconstruction claims must therefore be interpreted relative to the perturbation domain D. A model that achieves functional equivalence within the experimental domain may still fail when exposed to perturbations outside that domain.

This observation motivates the notion of domain-specific reconstructability. Let $D_{1}$ and $D_{2}$ denote two distinct perturbation domains. A behavioral system may be reconstructable within one domain but not the other if the perturbations available in the second domain fail to reveal critical aspects of the system dynamics (Strogatz, 2024). Reconstruction is therefore not an absolute property of a behavioral system but a relational property defined with respect to the experimental environment and the chosen model class.

These considerations lead to an important conceptual conclusion: reconstruction failures are themselves scientifically informative. If no model within a candidate class achieves functional equivalence under the admissible perturbations, this indicates that either the model class lacks sufficient expressive power or that the perturbation environment does not sufficiently probe the behavioral dynamics. In both cases, the failure provides guidance for refining the modeling framework or redesigning the experimental environment.

The study of identifiability and reconstructability limits therefore plays a central role in the proposed measurement paradigm. Rather than assuming that behavioral systems can always be faithfully modeled, the framework explicitly characterizes the conditions under which reconstruction is feasible and the circumstances under which it fails. This perspective transforms reconstruction from a purely methodological task into a scientific inquiry into the structural properties of behavioral systems and the experimental environments used to interrogate them.

## Special case: linear Gaussian behavioral systems

The reconstruction framework developed in the previous sections was formulated at a high level of generality, treating behavioral systems as stochastic controlled dynamical systems whose trajectory distributions must be inferred from perturbation–response data. While this general formulation allows for a wide range of non-linear and stochastic models, it is useful to consider specific model classes in which reconstruction can be analyzed more concretely. Linear Gaussian state-space systems provide a particularly instructive example because they combine analytical tractability with sufficient expressive power to capture a variety of behavioral dynamics (Kitagawa and Gersch, 1996).

In a linear Gaussian behavioral system, the latent behavioral state evolves according to a linear dynamical equation driven by external perturbations and stochastic noise. Let $x_{t}\in {\mathbb{R}}^{n}$ denote the latent state vector and $u_{t}\in {\mathbb{R}}^{m}$ the perturbation applied at time *t*. The system dynamics are described by


$$\begin{array}{l} x_{t+1}=Ax_{t}+Bu_{t}+w_{t}, \end{array}$$

where *A* is an *n*×*n* state transition matrix, *B* is an *n*×*m* perturbation coupling matrix, and *w*_*t*_ is a Gaussian process noise term with distribution


$$w_{t}\sim N(0,Q).$$

The observable behavioral output $y_{t}\in {\mathbb{R}}^{p}$ is generated from the latent state through the observation equation


$$\begin{array}{l} y_{t}=Cx_{t}+v_{t}, \end{array}$$

where *C* is a *p*×*n* observation matrix and *v*_*t*_ represents measurement noise with distribution


$$v_{t}\sim N(0,R).$$

Together, these equations define a stochastic linear dynamical system characterized by the parameter set


$$\begin{array}{l} \theta =\left(A,B,C,Q,R\right). \end{array}$$

Within this model class, perturbations influence behavioral trajectories through the matrix, which determines how environmental interventions affect the evolution of the latent behavioral state. The matrices *A* and *Q* describe the intrinsic dynamics of the behavioral system, including stability properties and stochastic fluctuations, while *C* and *R* determine how latent states are reflected in observable behavioral signals.

The reconstruction problem in this setting reduces to estimating the parameter set θ from perturbation–response trajectories. Given a dataset of observed trajectories and perturbation sequences


$$\begin{array}{l} D_{N}={\left\{\left(u_{0:T_{i}-1}^{\left(i\right)},y_{0:T_{i}}^{\left(i\right)}\right)\right\}}_{i=1}^{N}, \end{array}$$

parameter estimation can be performed through likelihood maximization or Bayesian inference. Because the system is linear and Gaussian, the likelihood of each trajectory can be computed efficiently using Kalman filtering and smoothing algorithms. These methods provide estimates of the latent state sequence as well as the parameters governing the system dynamics.

Within the linear Gaussian framework, the distribution of behavioral trajectories can be derived analytically. For a given perturbation sequence *u*_0:*T*−1_, the trajectory *y*_0:*T*_ follows a multivariate Gaussian distribution whose mean and covariance are determined by the system matrices and noise covariances. This analytical structure allows discrepancies between the behavioral system and reconstructed models to be evaluated using closed-form expressions for trajectory distributions. Perturbations play a particularly transparent role in this model class. Because the latent dynamics evolve linearly with respect to *u*_*t*_, different perturbation sequences produce predictable changes in the mean trajectory of the behavioral system. If the perturbation matrix *B* has full rank and the perturbation sequences explore sufficiently diverse directions in the perturbation space, the resulting trajectories provide strong constraints on the system parameters. In contrast, if perturbations remain confined to a low-dimensional subspace, certain parameters may become unidentifiable because different parameter configurations can produce identical observable trajectories (Ljung, 2010).

These considerations illustrate how perturbation design interacts with identifiability even in relatively simple dynamical systems. For example, consider the controllability matrix


$$\begin{array}{l} C=\left[\begin{array}{c} B\;AB\;A^{2}B\;\ldots \;A^{n-1}B \end{array}\right]. \end{array}$$

If this matrix has full rank, the perturbation inputs can influence all dimensions of the latent state space. In such cases, perturbations provide a powerful mechanism for probing the internal dynamics of the system. Conversely, if the controllability matrix is rank-deficient, certain latent dimensions remain inaccessible to perturbation, limiting the ability to reconstruct the corresponding behavioral dynamics.

The linear Gaussian model also allows a clear illustration of the concept of functional equivalence introduced earlier. Suppose a reconstructed model $M_{\hat{\theta }}$ has parameter estimates $Â,\hat{B},Ĉ,\hat{Q},\hat{R}$. For any perturbation sequence *u*_0:*T*−1_, the model generates a predicted trajectory distribution


$$P_{M_{\hat{\theta }}}(y_{0:T}|u_{0:T-1})=N(\mu_{\hat{\theta }},\Sigma_{\hat{\theta }}),$$

where $\mu_{\hat{\theta }}$ and $\Sigma_{\hat{\theta }}$ denote the mean and covariance implied by the estimated parameters. Functional equivalence can then be evaluated by comparing these predicted distributions to those generated by the behavioral system under novel perturbations.

Because Gaussian distributions admit closed-form divergence measures, discrepancies between trajectory distributions can be computed analytically. For example, the Wasserstein distance between two Gaussian trajectory distributions depends only on the differences between their means and covariance matrices (Peyré and Cuturi, 2019). This property enables efficient evaluation of functional equivalence across large sets of perturbation trajectories.

Although linear Gaussian systems represent a simplified model class, they provide an instructive bridge between the abstract reconstruction framework and practical behavioral modeling. Many existing approaches to modeling cognitive and affective dynamics can be expressed as special cases of linear state-space systems or as non-linear extensions thereof (Waugh and Kuppens, 2021; Valenza et al., 2011; Markov et al., 2015; Hollenstein and Lewis, 2006; Hamaker et al., 2015). Moreover, the analytical tools developed for linear dynamical systems—such as controllability analysis, Kalman filtering, and optimal experiment design—provide a foundation for implementing perturbation-based behavioral reconstruction in real experimental settings.

More broadly, the linear Gaussian case illustrates how the general reconstruction framework can be instantiated within concrete model classes. By analyzing identifiability, perturbation informativeness, and functional equivalence within specific dynamical models, researchers can develop practical reconstruction strategies tailored to particular behavioral domains.

The considerations above suggest that reconstruction critically depends on the informativeness of perturbations. This dependence can be made explicit in a simple controlled setting, where identifiability conditions can be directly observed.

## Simulation

To demonstrate the practical feasibility of the proposed framework, we consider a simple linear Gaussian dynamical system with known parameters, where behavioral dynamics are generated according to a controlled state-space model and perturbations act as external inputs.

A dataset of perturbation–response trajectories is generated under two distinct conditions: (i) a rich perturbation regime with sufficient variability, and (ii) a low-variability regime with nearly constant inputs. In both cases, models are estimated from the training data using standard system identification techniques and subsequently evaluated under a set of unseen test perturbations (See Appendix A).

**Figure 2:**
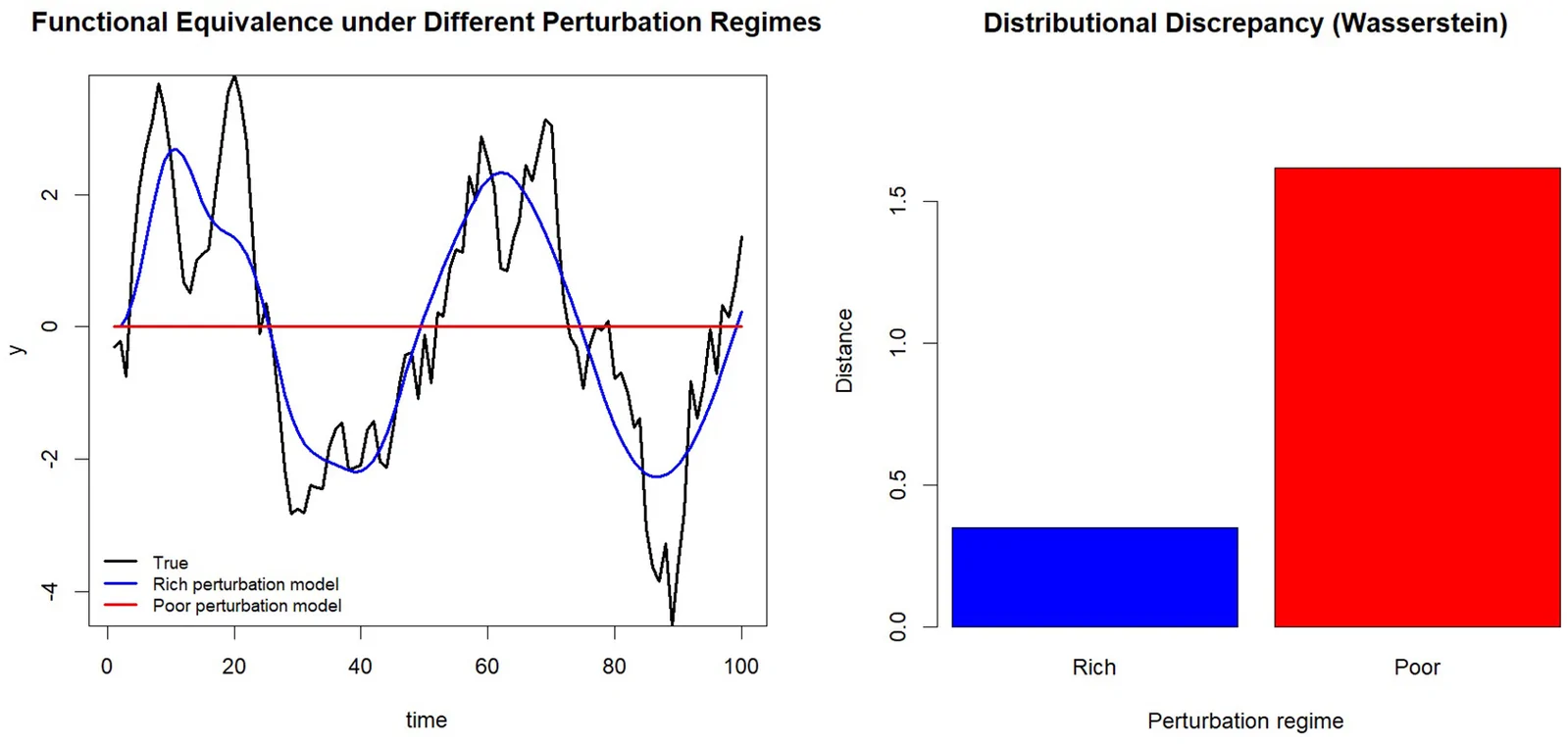
Functional equivalence under different perturbation regimes. **(Left)** Example trajectories generated by the true system (black), the model reconstructed under rich perturbations (blue), and the model reconstructed under poor perturbations (red), evaluated under an unseen test input. **(Right)** Distributional discrepancy (Wasserstein distance) between true and model-generated trajectories. Rich perturbations yield low discrepancy, while poor perturbations lead to substantial deviations, highlighting the role of perturbation informativeness in behavioral system reconstruction. Numerical values: Wasserstein distance = 0.35 (rich) vs. 1.62 (poor).

Functional equivalence is assessed by comparing empirical trajectory distributions obtained from the true system and from the reconstructed model using a Wasserstein discrepancy measure. Results show a clear divergence between the two regimes (Figure 2). Under rich perturbations, the reconstructed model produces trajectories that closely match the true system dynamics, resulting in low discrepancy and statistical indistinguishability across test conditions. In contrast, under low-variability perturbations, the reconstruction fails to capture the system behavior, leading to substantially higher discrepancy and degenerate trajectory predictions.

These findings demonstrate that the success of behavioral system reconstruction critically depends on the informativeness of the perturbations. In the absence of sufficiently rich inputs, the estimation problem becomes not only inaccurate but effectively ill-posed, as the available data do not contain enough information to identify the underlying system.

## Behavioral Domains and Empirical Instantiation

Human behavioral processes manifest across diverse domains characterized by different dynamical structures, temporal scales, and forms of environmental interaction. Emotional regulation, cognitive flexibility, stress reactivity, and social coordination all involve behavioral states that evolve over time and respond to external perturbations. Within the reconstruction framework introduced in this paper, these domains can be conceptualized as stochastic dynamical systems whose internal states are probed through controlled perturbations and whose dynamics are inferred from the resulting behavioral trajectories. Crucially, perturbations are not introduced merely as experimental stimuli but as interventions designed to induce transitions between latent behavioral states, thereby revealing the structure of the underlying system dynamics (Strogatz, 2024). Different behavioral domains therefore require perturbation environments capable of eliciting domain-relevant state transitions while producing trajectories that can be modeled using suitable dynamical representations. Table 1 provides illustrative examples linking behavioral domains, targeted state transitions, perturbation environments, and candidate model classes that may be used to reconstruct the corresponding behavioral dynamics (Kloeden and Pearson, 1977; Platen and Bruti-Liberati, 2010; Ghahramani and Hinton, 1996; Eddy, 2004; Wiggins, 2003; Ghaffarzadegan et al., 2024; Durbin and Koopman, 2012; Gilbert, 2019).

**Table 1:** Illustrative instantiations of perturbation-based behavioral reconstruction.

Behavioral domain	Targeted state transitions	Perturbation environment	Example VR manipulation	Observable behavioral trajectories	Illustrative model classes
Affective dynamics	Transitions between emotional regimes (e.g., relaxation → stress → recovery)	Emotional perturbation protocol	VR scenarios manipulating social evaluation, environmental threat, or calming environments	Continuous affect ratings, heart-rate variability, gaze dynamics, body movement	Stochastic differential equations; non-linear dynamical systems
Cognitive flexibility	Transitions between task strategies or cognitive control states	Adaptive task environment	VR tasks with rule switches, changing contingencies, or unexpected feedback	Decision sequences, response times, error dynamics	Hidden Markov models; switching state-space models
Stress reactivity and resilience	Transitions between stable performance and breakdown under stress	Escalating challenge environment	Increasing time pressure, environmental noise, or unpredictable events	Performance degradation trajectories, physiological stress indicators, recovery dynamics	Non-linear dynamical systems; stochastic stability models
Social interaction dynamics	Transitions between coordination patterns among interacting agents	Multi-agent interaction environment	Cooperative or competitive VR scenarios with adaptive agent behavior	Joint behavioral trajectories, communication timing, coordination metrics	Agent-based models; coupled dynamical systems

A first class of behavioral systems concerns affective dynamics. Emotional states evolve continuously over time and are influenced by both external events and internal regulatory processes. Let *x*_*t*_ represent a low-dimensional latent state describing the affective configuration of an individual at time *t*. Environmental events, social interactions, or cognitive demands may act as perturbations *u*_*t*_ that modify this state. Observable outputs *y*_*t*_ may include self-reported affect ratings, physiological measures such as heart rate variability, or behavioral indicators such as facial expression and movement patterns.

Within this domain, reconstruction seeks to identify dynamical models capable of capturing how affective states evolve under perturbations and how individuals recover from disturbances. Perturbation environments may involve controlled emotional stimuli, social feedback manipulations, or task demands designed to induce fluctuations in affective states (Ljung, 2010). The resulting trajectory data allow researchers to estimate dynamical properties such as stability, inertia, and resilience of affective systems.

A second important domain concerns cognitive flexibility and adaptive behavior. Cognitive systems must constantly adapt to changing environmental demands, switching strategies when task contingencies change. In such contexts, perturbations may correspond to shifts in task rules, introduction of conflicting stimuli, or modifications of reward structures. The latent behavioral state may represent internal representations of task structure, cognitive control allocation, or belief states about environmental contingencies.

Reconstruction in this domain aims to identify models capable of reproducing how individuals adapt to perturbations in task structure. Behavioral trajectories may include sequences of decisions, response times, and action patterns that evolve as individuals learn or adapt to new conditions. By systematically manipulating environmental contingencies, perturbation environments can reveal how cognitive systems transition between stable strategies and how they recover from disruptive changes.

A third domain involves stress reactivity and resilience. Human behavioral systems exhibit complex responses to environmental stressors, including physiological, cognitive, and affective components. Perturbations in this context may involve time pressure, uncertainty, social evaluation, or unexpected environmental disruptions. Observable outputs may include behavioral performance, physiological stress indicators, and patterns of decision making.

Within the reconstruction framework, such perturbations allow researchers to probe the stability boundaries of behavioral systems. Mild perturbations may produce only transient deviations from baseline behavior, while stronger perturbations may push the system toward non-linear transitions or breakdowns in adaptive functioning. By analyzing how behavioral trajectories evolve under increasing perturbation intensity, reconstruction methods can estimate resilience properties of the system and identify critical transition points.

Social interaction dynamics provide another domain in which the reconstruction paradigm becomes particularly relevant. Social behavior often involves coupled dynamical systems in which multiple agents interact and influence one another. The behavioral state of each participant evolves not only in response to environmental perturbations but also in response to the behavior of other agents. Perturbations in this context may involve modifications of interaction rules, introduction of cooperative or competitive incentives, or changes in communication channels.

In such systems, reconstruction must account for the mutual influence between interacting agents. Behavioral trajectories become joint trajectories describing the evolution of multiple behavioral states over time. Identifying the underlying dynamical structure requires perturbation environments capable of manipulating interaction structures while recording the resulting behavioral responses of all participants.

Across these domains, the reconstruction framework provides a unifying perspective on behavioral measurement. Rather than treating emotional states, cognitive strategies, or social behaviors as isolated variables, the framework treats them as dynamical systems whose properties can be inferred from perturbation–response trajectories. Different domains may require different model classes and perturbation environments, but the underlying methodological principle remains the same: behavioral systems are reconstructed by systematically probing their responses to controlled interventions.

Virtual environments and programmable experimental platforms play a crucial role in enabling such empirical instantiations. These environments allow perturbations to be implemented along multiple dimensions simultaneously, including sensory, cognitive, and social variables. At the same time, they provide high-resolution measurements of behavioral trajectories through multimodal sensing technologies. The integration of controlled perturbations with continuous behavioral measurement creates the conditions necessary for applying the reconstruction methodology across diverse behavioral domains.

Importantly, the framework does not assume that all behavioral systems are equally reconstructable. Different domains may exhibit varying degrees of dynamical complexity and stochastic variability. Some behavioral processes may be well-approximated by relatively simple dynamical models, while others may require highly non-linear or high-dimensional representations. By applying the reconstruction framework across multiple behavioral domains, researchers can empirically investigate the limits of reconstructability and identify which aspects of human behavior can be faithfully modeled within bounded experimental environments.

In this way, the empirical instantiation of the reconstruction framework serves not only to model specific behavioral systems but also to advance a broader scientific objective: understanding the extent to which human behavior can be represented as a generative dynamical process whose observable trajectories can be reproduced under controlled perturbations.

## Relation to psychometrics and computational modeling

The reconstruction framework proposed in this work occupies an intermediate position between two established traditions in behavioral science: classical psychometrics and contemporary computational modeling (Marsman et al., 2023). While both traditions aim to formalize and explain behavioral data, they differ substantially in their assumptions about what constitutes a measurement and how behavioral systems should be represented. The present framework integrates elements from both perspectives while extending them toward a dynamical and intervention-based conception of behavioral measurement.

Classical psychometrics has historically focused on the measurement of latent traits inferred from patterns of observed responses. In this paradigm, behavioral observations are typically treated as manifestations of stable underlying attributes such as abilities, attitudes, or personality traits. Measurement models, such as factor analysis or item response theory, establish probabilistic relationships between latent variables and observed responses, allowing the estimation of latent scores from observed data. These approaches have proven extremely powerful for constructing reliable instruments and for analyzing large-scale behavioral datasets. However, they generally assume that the underlying latent structure is static or slowly varying, and they rely on repeated observations of similar stimuli rather than on controlled perturbations of the behavioral system.

The reconstruction framework introduced in this paper adopts a different perspective on behavioral measurement. Instead of treating latent variables as static attributes inferred from aggregated responses, it treats behavior as the observable output of an evolving dynamical system. Within this view, measurement consists not merely of estimating latent scores but of identifying the generative mechanisms that produce behavioral trajectories under controlled perturbations. Observable data are therefore interpreted as realizations of a perturbation-response mapping that reflects the internal dynamics of the behavioral system. The object of measurement becomes the trajectory law induced by the system rather than a static parameter associated with an individual.

This shift from latent trait estimation to dynamical reconstruction does not invalidate the contributions of classical psychometrics. On the contrary, many psychometric models can be interpreted as special cases of the broader dynamical framework considered here. For example, item response models may be viewed as simplified observation models in which latent variables remain constant during the measurement process and perturbations correspond to the presentation of test items. In this sense, psychometric measurement can be understood as a limiting case of behavioral reconstruction in which the temporal dimension and dynamical interactions are suppressed. Extending psychometric models to incorporate temporal dynamics, state transitions, and controlled perturbations therefore represents a natural generalization of existing measurement theory.

At the same time, the proposed framework connects naturally with recent developments in computational modeling of behavior. Over the past two decades, computational approaches have increasingly represented cognitive and behavioral processes using dynamical systems, reinforcement learning models, Bayesian inference mechanisms, and agent-based simulations. These models aim to capture the generative processes underlying behavior rather than merely describing statistical associations among observed variables. Computational modeling therefore shares with the reconstruction framework the goal of explaining behavior in terms of mechanistic processes.

However, computational models are often developed primarily as theoretical constructs or explanatory tools rather than as measurement instruments. Model evaluation typically relies on predictive accuracy or goodness-of-fit metrics applied to observational data. While these criteria are useful for comparing models, they do not necessarily establish that a model faithfully reproduces the behavioral dynamics of the system under novel conditions. The reconstruction framework addresses this limitation by introducing the criterion of functional equivalence under controlled perturbations. A reconstructed model is considered valid only if it produces trajectory distributions indistinguishable from those generated by the behavioral system when exposed to previously unseen perturbations.

This emphasis on perturbation-based validation aligns the framework with principles from system identification and experimental control theory. In these disciplines, models are not evaluated solely on their ability to fit observed data but on their ability to reproduce the system's response to external inputs across a range of conditions. By adopting a similar perspective, behavioral reconstruction transforms experimental environments into identification instruments capable of probing the internal dynamics of human behavior.

More fundamentally, in the absence of sufficiently informative perturbations, the reconstruction problem becomes not only less accurate but effectively ill-posed, as the available data do not contain enough information to identify the underlying system dynamics.

The resulting perspective suggests a synthesis of psychometric measurement and computational modeling. Psychometrics contributes rigorous statistical frameworks for inference and uncertainty quantification, while computational modeling provides mechanistic representations of behavioral processes. The perturbation-based reconstruction framework integrates these traditions within a unified methodological structure in which behavioral systems are interrogated through controlled interventions and reconstructed as generative dynamical models. This integration opens the possibility of developing measurement instruments that do not merely estimate latent traits but actively reconstruct the dynamical mechanisms governing human behavior.

## Toward a metrology of human behavior

The framework developed in this paper suggests a broader conceptual shift in how behavioral measurement can be understood. Traditionally, the measurement of psychological constructs has relied on statistical associations between observed responses and latent variables. Although these approaches have produced powerful tools for assessing cognitive abilities, personality traits, and attitudes, they rarely establish measurement in the strict sense used in the physical sciences. In most cases, behavioral constructs are inferred from patterns of responses rather than directly tied to experimentally controlled transformations of the system under study. The perturbation-based reconstruction framework opens the possibility of moving toward a more rigorous conception of behavioral measurement that parallels the logic of metrology.

In the physical sciences, metrology refers to the science of measurement and involves the systematic definition of quantities, units, and measurement procedures that yield reproducible and comparable results across laboratories and contexts. Measurements are typically grounded in controlled interactions between a measuring instrument and the physical system of interest. Through these interactions, observable responses reveal properties of the underlying system, allowing researchers to estimate quantities that characterize its structure or dynamics. The validity of a measurement procedure is established by demonstrating that it reliably captures invariant properties of the system across repeated observations and varying experimental conditions.

A similar perspective can be adopted for the study of human behavior. Within the reconstruction framework, experimental environments (such as adaptive tasks, immersive virtual reality scenarios, or interactive social simulations), function as measurement instruments designed to probe the dynamics of behavioral systems. Controlled perturbations serve the role of measurement operations, systematically interacting with the behavioral system and eliciting responses that reveal its internal structure. Observable trajectories, such as sequences of actions, physiological responses, or interaction patterns, correspond to the measurement signals generated by these operations.

From this viewpoint, behavioral measurement no longer consists solely of assigning numerical scores to individuals but of identifying the dynamical mechanisms that govern behavioral responses to perturbations. The quantities of interest are therefore not limited to static latent variables but may include dynamical parameters, transition structures, stability properties, or other characteristics of the underlying behavioral system. In this sense, behavioral reconstruction aims to estimate the generative laws that determine how behavior evolves under controlled environmental inputs.

An important implication of this perspective concerns the role of experimental design. In classical psychometric testing, items are selected primarily to maximize reliability or discriminative power. In a metrological framework, perturbations must instead be designed to maximize the identifiability of the behavioral system. Different perturbation policies may reveal different aspects of the system's dynamics, just as different experimental probes in physics can reveal complementary properties of a physical system. The design of perturbation environments thus becomes an integral part of the measurement process rather than a secondary consideration.

Another key element of metrology is the notion of reproducibility across instruments and laboratories. For behavioral measurement, this requirement implies that reconstructed models should produce consistent trajectory laws when behavioral systems are probed under equivalent perturbation policies. Achieving such reproducibility will likely require the development of standardized perturbation protocols, shared experimental environments, and common data representations that allow results to be compared across studies. Advances in immersive technologies and digital experimental platforms make such standardization increasingly feasible.

The metrological perspective also highlights the importance of validation procedures that go beyond conventional goodness-of-fit metrics. In the present framework, the criterion of functional equivalence under unseen perturbations plays a central role. A reconstructed model is considered an adequate representation of the behavioral system if it generates trajectory distributions that match those produced by the real system when exposed to novel perturbation sequences. This requirement ensures that the reconstructed model captures not only statistical regularities in observed data but also the underlying dynamical principles governing behavioral responses.

Developing a full metrology of human behavior remains an ambitious goal. It will require integrating advances from several disciplines, including psychometrics, dynamical systems theory, computational modeling, experimental design, and human–computer interaction. Nevertheless, the perturbation-based reconstruction framework provides a conceptual foundation for such an endeavor. By treating behavioral environments as measurement instruments and by focusing on the reconstruction of generative dynamics, it opens a path toward a more principled and experimentally grounded science of behavioral measurement.

## Challenges

The perturbation-based reconstruction framework outlined in this paper suggests that behavioral measurement may be redefined as the identification of dynamical systems through controlled interaction with experimental environments. While this perspective opens new methodological possibilities, it also raises a set of fundamental challenges that must be addressed for a fully developed science of behavioral measurement to emerge.

A first challenge concerns the design of perturbation environments capable of efficiently probing behavioral systems. The informativeness of reconstruction depends critically on the structure of the perturbations used to interrogate the system. Designing perturbation policies that maximize discriminability between competing models while remaining ecologically meaningful and ethically acceptable remains an open problem. This challenge becomes even more complex in adaptive settings, where perturbations evolve in response to ongoing behavior and must balance exploration and stability.

A second challenge relates to the standardization of behavioral measurement procedures. In physical metrology, measurement depends on well-defined instruments and protocols that ensure reproducibility across laboratories. Achieving a comparable level of standardization in behavioral science will require the development of shared perturbation protocols, common experimental platforms, and interoperable data representations. Programmable environments, including immersive and VR-based systems, provide a potential infrastructure for such standardization, but their widespread adoption raises issues of comparability, calibration, and validation across different implementations.

A third challenge concerns the identifiability and scalability of behavioral system reconstruction. Human behavior is inherently high-dimensional, stochastic, and context-dependent. Developing model classes that are sufficiently expressive to capture this complexity while remaining identifiable from finite data represents a major methodological problem. In addition, reconstruction methods must scale to large populations while preserving sensitivity to individual differences in dynamical structure.

A fourth challenge involves the integration of multimodal behavioral data. Advances in sensing technologies enable the simultaneous recording of movement, physiology, gaze, and interaction patterns, generating high-dimensional trajectory data. While such data increase observability, they also introduce challenges related to data integration, noise modeling, and computational complexity. Establishing principled methods for combining multimodal signals into coherent dynamical representations of behavior remains an open research direction.

A fifth challenge concerns the role of artificial agents and generative models in behavioral experimentation. The emergence of generative artificial intelligence makes it possible to construct adaptive environments in which perturbations are dynamically generated in response to participant behavior. Such systems can potentially enhance the efficiency of system identification by actively exploring informative regions of the behavioral state space. However, they also raise questions about interpretability, control, and the extent to which artificially generated interactions faithfully represent real-world behavioral contexts.

A sixth challenge relates to the definition of behavioral quantities and units within a metrological framework. In the physical sciences, measurement is grounded in well-defined units and invariant transformations. In behavioral science, the corresponding quantities remain less clearly defined. If behavioral systems are to be measured as dynamical entities, it becomes necessary to identify which aspects of their dynamics constitute meaningful and comparable quantities, such as stability, flexibility, or resilience, and how these can be operationalized across different experimental settings.

Finally, a broader conceptual challenge concerns the integration of this framework within the existing landscape of behavioral science. The perturbation-based perspective does not replace established approaches such as psychometrics or computational modeling, but rather extends them toward a more dynamical and experimentally grounded conception of measurement. Developing a coherent synthesis that preserves the strengths of existing methodologies while enabling system-level reconstruction will be essential for the adoption of this paradigm.

Addressing these challenges will require coordinated advances in experimental design, computational modeling, measurement theory, and technological infrastructure. While substantial work remains, the convergence of programmable environments, multimodal sensing, and generative modeling suggests that the conditions necessary for a new science of behavioral measurement are beginning to emerge.

## Discussion

The framework proposed in this paper suggests a shift in how behavioral phenomena may be conceptualized and measured within the behavioral sciences. Rather than treating behavior primarily as the manifestation of static latent traits, the perturbation-based reconstruction perspective conceptualizes behavior as the observable output of an evolving dynamical system. Within this view, measurement consists not only in estimating latent quantities but in reconstructing the generative mechanisms that govern behavioral responses to controlled environmental perturbations.

This perspective places behavioral science more closely with disciplines in which system identification plays a central methodological role. In fields such as physics, engineering, and control theory, systems are studied by systematically probing them with controlled inputs and analyzing the resulting trajectories. The goal is not merely to describe observations but to infer the underlying dynamical structure capable of generating those observations. The framework developed here extends this logic to the study of human behavior by interpreting experimental environments as instruments that interact with behavioral systems through structured perturbations.

Historically, such an approach has been difficult to implement in the behavioral sciences. Experimental paradigms have typically faced a trade-off between ecological validity and experimental control. Laboratory tasks provide precise manipulation and repeatability but often capture only simplified aspects of behavior, while naturalistic observations and experience sampling methods offer ecological realism but limited control over the perturbations experienced by participants. As a consequence, most measurement frameworks have focused on aggregating responses into stable scores rather than reconstructing the underlying behavioral dynamics that generate those responses.

Recent technological developments are beginning to change this situation. Immersive experimental environments, particularly those enabled by virtual reality, allow researchers to design perturbation protocols that are both systematically controllable and ecologically meaningful. Within such environments, the context, timing, and intensity of perturbations can be precisely manipulated while maintaining a level of experiential realism that approximates real-world situations. At the same time, advances in sensing technologies make it possible to record high-dimensional behavioral trajectories, including movement, gaze patterns, physiological responses, and interaction dynamics.

These developments create the experimental conditions required for perturbation-based reconstruction of behavioral systems. Virtual reality environments can function as programmable measurement platforms in which controlled environmental manipulations serve as probing signals, while multimodal behavioral recordings provide the observable trajectories from which the system's dynamics can be inferred. In this sense, immersive environments play a role analogous to experimental apparatus in the physical sciences: they enable systematic interactions between the measurement instrument and the system under investigation.

The emergence of generative artificial intelligence further amplifies these possibilities. Generative models and adaptive agents can be used to construct interactive environments in which perturbations evolve dynamically in response to the participant's behavior. This makes it possible to design adaptive perturbation policies that explore the behavioral system more efficiently, potentially improving the identifiability of the reconstructed dynamics. Moreover, artificial agents embedded in immersive environments can simulate complex social interactions, enabling the systematic study of behavioral systems that emerge from multi-agent dynamics.

Taken together, these technological and methodological developments suggest that behavioral science may be entering a phase in which the reconstruction of dynamical behavioral systems becomes experimentally feasible. The perturbation-based framework proposed in this work provides a conceptual structure for this transition by linking controlled perturbations, behavioral trajectories, and generative modeling within a unified measurement perspective.

Recent advances in generative and predictive modeling have demonstrated an increasing ability to simulate realistic behavioral trajectories. However, prediction alone does not constitute measurement. In the absence of controlled perturbations and validation under novel conditions, such models may capture statistical regularities in observed data without identifying the underlying behavioral system. From this perspective, the distinction between predictive adequacy and measurement validity becomes central to the development of a metrology of human behavior.

At the same time, several challenges remain before a full metrology of human behavior can be realized. The design of perturbation policies that efficiently probe behavioral systems remains an open problem. Standardized experimental environments and perturbation protocols will likely be required to ensure reproducibility across laboratories. In addition, the development of model classes capable of capturing the complexity of human behavior while remaining identifiable from empirical data represents an important methodological challenge.

Despite these challenges, the perturbation-based reconstruction framework highlights a promising direction for the future of behavioral measurement. By treating experimental environments as instruments, perturbations as measurement operations, and behavioral trajectories as dynamical signals, it opens the possibility of moving beyond static representations of behavior toward the identification of generative behavioral systems. Such a shift could provide a foundation for a more principled and experimentally grounded science of human behavior.

## Conclusions

In this perspective, the measurement of human behavior may evolve from the estimation of latent traits toward the identification of dynamical behavioral systems. Rather than treating behavioral observations as isolated responses to predefined stimuli, the perturbation-based reconstruction framework interprets them as trajectories generated by an underlying system interacting with its environment. Measurement thus becomes an active process in which experimental environments are designed to probe the structure of behavioral dynamics through controlled perturbations.

Advances in immersive environments, sensing technologies, and computational modeling now make it possible to implement this approach experimentally. Virtual reality environments can function as programmable measurement platforms in which complex yet controllable perturbations are applied to behavioral systems, while multimodal sensing allows the continuous observation of behavioral trajectories. When combined with generative models and adaptive perturbation strategies, these environments enable a form of experimental probing that was previously unattainable in behavioral research.

The framework proposed here therefore points toward the possibility of a more principled approach to behavioral measurement grounded in system identification. By integrating controlled perturbations, dynamical modeling, and trajectory-level validation, it provides a conceptual foundation for reconstructing the generative mechanisms underlying human behavior. Although substantial methodological challenges remain, the convergence of immersive technologies, computational modeling, and experimental design suggests that the reconstruction of behavioral dynamics may become a central methodological paradigm for future behavioral science. More broadly, this perspective reframes behavioral measurement as the problem of identifying generative systems through controlled interactions, bringing it conceptually closer to the measurement traditions of the physical sciences while preserving the complexity of human behavior.

In this sense, the long-standing ambition of developing a rigorous measurement science of human behavior, analogous in spirit to metrology in the physical sciences, may begin to move from conceptual aspiration toward practical possibility.

## Data Availability

The original contributions presented in the study are included in the article/Supplementary material, further inquiries can be directed to the corresponding author.

## Appendix

**Appendix A**. Simulation procedure. The following pseudo-code illustrates the core steps of the simulation and reconstruction procedure used in the study. Full implementation details are provided in the Supplementary materials (**R code**). *Perturbation-based behavioral system reconstruction (simulation)* Input: System parameters (A, B, C), noise models, perturbation regimes Output: Reconstructed model M and discrepancy measures

1. Define ground-truth dynamical system

x_{t+1}=Ax_t+Bu_t+w_ty_t=Cx_t+v_t

2. Generate perturbation sequences a. Rich regime: u_t distribution with sufficient variability b. Poor regime: u_t≈ constant or low-variance process
3. Simulate trajectory dataset For each trajectory i = 1, …, N : Generate (x_t^∧^{(i)}, y_t^∧^{(i)}) under chosen perturbation regime
4. Estimate model from data Fit parameters (Â, B) using system identification on training trajectories
5. Evaluate on unseen perturbations Generate new input sequence u_test Simulate trajectories from both true system and reconstructed model
6. Assess functional equivalence Compute discrepancy D between trajectory distributions (e.g., Wasserstein distance)
7. Compare regimes Analyze D under rich vs. poor perturbations

Return: Estimated model and discrepancy measures
